# Beverage consumption habits “24/7” among British adults: association with total water intake and energy intake

**DOI:** 10.1186/1475-2891-12-9

**Published:** 2013-01-10

**Authors:** Sigrid Gibson, Susan M Shirreffs

**Affiliations:** 1 Sig-Nurture Ltd., Guildford, Surrey Gu1 2TF, UK; 2 School of Sport, Exercise and Health Sciences, Loughborough University, Loughborough, LE11 3TU, UK

**Keywords:** Water intake, Energy, Beverage consumption, Adults, Dietary patterns

## Abstract

**Background:**

Various recommendations exist for total water intake (TWI), yet it is seldom reported in dietary surveys. Few studies have examined how real-life consumption patterns, including beverage type, variety and timing relate to TWI and energy intake (EI).

**Methods:**

We analysed weighed dietary records from the National Diet and Nutrition Survey of 1724 British adults aged 19–64 years (2000/2001) to investigate beverage consumption patterns over 24 hrs and 7 days and associations with TWI and EI. TWI was calculated from the nutrient composition of each item of food and drink and compared with reference values.

**Results:**

Mean TWI was 2.53 L (SD 0.86) for men and 2.03 L (SD 0.71) for women, close to the European Food Safety Authority “adequate Intake” (AI) of 2.5 L and 2 L, respectively. However, for 33% of men and 23% of women TWI was below AI *and* TWI:EI ratio was <1 g/kcal. Beverages accounted for 75% of TWI. Beverage variety was correlated with TWI (r 0.34) and more weakly with EI (r 0.16). Beverage consumption peaked at 0800 hrs (mainly hot beverages/ milk) and 2100 hrs (mainly alcohol). Total beverage consumption was higher at weekends, especially among men. Overall, beverages supplied 16% of EI (men 17%, women 14%), alcoholic drinks contributed 9% (men) and 5% (women), milk 5-6%, caloric soft drinks 2%, and fruit juice 1%.

In multi-variable regression (adjusted for sex, age, body weight, smoking, dieting, activity level and mis-reporting), replacing 100 g of caloric beverages (milk, fruit juice, caloric soft drinks and alcohol) with 100 g non-caloric drinks (diet soft drinks, hot beverages and water) was associated with a reduction in EI of 15 kcal, or 34 kcal if food energy were unchanged. Using within-person data (deviations from 7-day mean) each 100 g change in caloric beverages was associated with 29 kcal change in EI or 35 kcal if food energy were constant. By comparison the calculated energy content of caloric drinks consumed was 47 kcal/100 g.

**Conclusions:**

TWI and beverage consumption are closely related, and some individuals appeared to have low TWI. Energy from beverages may be partly compensated. A better understanding of interactions between drinking and eating habits and their impact on water and energy balance would give a firmer basis to dietary recommendations.

## Introduction

Water is arguably the most critical nutrient, as its absence can be lethal within a few days [1-3]. To promote adequate water intake at the population level, several countries and transnational authorities have developed water intake recommendations based on national estimates of water intake [4-6]. In the UK, data on total water intake (TWI) is not normally published in national survey reports and there is currently no Dietary Reference Value. The main purpose of this study was to quantify TWI and its relation to patterns of beverage consumption and then to explore associations between types of beverage consumed and the intake of water and energy. It has been estimated from studies in Europe and the USA that around 70% -80% of TWI comes from beverages of various types (including water, tea and coffee, milk, soft drinks, juice and alcoholic drinks), with the remainder contributed by water in food [4,7], and we expected the British population would have a similar pattern. Beverages are widely available in developed societies, where tap water is essentially free, but there are nevertheless concerns that some people may not be consuming sufficient fluid for optimal health and some people may be over-consuming. Children, elderly or infirm people, and those working in hot environments, are some of those most vulnerable to the effects of dehydration, but many adults may also be inadequately hydrated at some time. Unfortunately, hydration status is rarely measured in epidemiological studies and this hampers attempts to assess the adequacy of water intakes at a population level. However, guidelines have been established to determine how much water humans require (on average) to avoid dehydration and to optimise physical and psychological function.

In 2005, the Food and Nutrition board of the Institute of Medicine (IOM) published adequate intake (AI) values for TWI in temperate climates [5]. The AI for total water (from a combination of drinking water, beverages, and food) was set based on the median total water intake from US survey data. For young men and women (19–30 years) this is 3.7 L and 2.7 L per day, respectively. These recommended intakes are based on median intakes of generally healthy individuals who are adequately hydrated, but the report pointed out that individuals can be adequately hydrated at levels below, as well as above, the AIs provided. The American AIs exceed those of other authorities, while the recent recommendations produced by the European Food Safety Authority (EFSA) in 2010 are the most conservative to date, at 2.0 L per day for adult females and 2.5 L per day for adult males [4].

Limited data exist on daily water intake in other countries, and comparison is sometimes hampered by different methods of definition and data collection [8]. In the UK, TWI is not currently quoted in the published reports but the mean value has been calculated as 2494 g/day among adults aged 19–64 y in 2008/9 [9]. Average water intakes in other European countries appear to be broadly similar to those in the UK (e.g. mean 2461 ml/day in Sweden [10]), or else lower than the UK, (e.g. mean intake 1984 ml/d in France [11]; 2039 ml/day in Germany [12]; 2222 ml/d in the Netherlands [13]). Reported intakes of total water in North America are considerably higher than in Britain and Europe. In 2005–2006, American adults participating in the National Health and Nutrition Examination Surveys (NHANES) reported consuming 3.18 L of total water within the previous 24 hours [7], slightly less than the 3.35 L reported in 1999–2004.

While all beverages support hydration by virtue of their high water content, many also supply calories. Excessive consumption of caloric beverages has been widely viewed as contributing to the obesity epidemic [14,15], although systematic reviews have highlighted the need for better randomised controlled trials, in order to demonstrate a causal effect [16,17]. The main putative mechanism involves reduced satiety and incomplete compensation for calories ingested in liquid form [18]. According to this theory, so-called “liquid calories” are more likely than solid calories to result in passive overconsumption and excess energy intake [19]. There are, however, few studies that have examined relationships between beverage consumption patterns and energy intake in the British population, apart from the recent paper by Ng et al. [9], which reported that the proportion of energy from beverages changed very little between 1986 and 2008/9, although there were some shifts between sources. Our paper differs in scope, focusing on consumption over 24 hours and 7 days of the week and comparing water intakes, in men and women with reference values.

The National Diet and Nutrition Survey database provides what is probably the best source of detailed information on the diets of normal individuals in Britain [20,21]. In 2008 the survey adopted a new method, collecting data via a (non-weighed) diet record over 4 days, in place of the former weighed record over 7 days, in 2000/2001. The latter, slightly older, data provide the opportunity to study variation over days of the week and may give a better indication of participants’ usual intake. The food records list the weight in grams of each item of food and drink consumed (including tap water) for each of the 1724 participants (12,068 person-days of data), while the nutrient database includes the water, energy and nutrient content of each item. Time of consumption is also recorded, providing a rich resource for exploring patterns of consumption (timing, frequency, variety etc.) and linking this with personal data. Using raw data from the NDNS we attempted to address the following questions:

1) Is the UK adult population consuming adequate amounts of total water?

2) How does beverage consumption vary by age and gender, day of the week and time of day and is this related to total water intake?

3) Is the variety of beverages consumed a positive predictor of total water intake?

4) How much energy do beverages contribute to the UK diet and in what proportion?

5) Is energy in liquid form (i.e. from beverages) positively associated with total energy intake?

## Methods

### The survey

The *National Diet and Nutrition Survey: adults aged 19 to 64 years,* (NDNS) is a nationally representative survey of the diet and health of adults living in private households in Great Britain in 2000/2001 [21]. It was commissioned jointly by the Department of Health and the Food Standards Agency and ethical approval was obtained via the normal channels. Fieldwork was conducted over a 12-month period in 2000/2001 to cover any seasonality in eating behavior and in the nutrient content of foods. Overall, 61% of the eligible sample (n = 3704) completed the dietary interview (responding sample, n = 2,251) and 77% of those who completed the dietary interview completed a full seven-day weighed dietary record (diary sample, n = 1724, representing 12068 person-days of data) [21]. There was no evidence of serious non-response bias, although inevitably surveys of this type favour willing participants whose diets may be less extreme, or less variable day to day.

Respondents were asked to keep a weighed record of all food and drink consumed, both in and out of the home. Each respondent was issued with a set of digital food scales and instructed how to weigh and record items in two diaries, “Home” and “Eating out”. A description of each item was recorded, including the brand name of the product and if appropriate, the method of preparation. Also recorded was the time (to the nearest 5 minutes) and location. Everything consumed by the respondent had to be recorded, including drinks of water and medications and supplements. Respondents were asked to weigh everything they could as separate items in order that the nutrient composition of each could be calculated. Recipes for all home-made dishes were collected. Where it was not possible to weigh the food, respondents were asked to record as much information as possible, particularly the portion size and an estimate of any leftover and, for meals out, price and place of purchase. In certain circumstances duplicate portions were purchased for weighing. Interviewers visited workplace canteens to collect information on portion sizes, cooking methods and ingredients.

Each interviewer called back 24 hours after placing the diaries to check that the items were being recorded correctly, to give encouragement and re-motivate where appropriate. Further visits were included as necessary and any apparent omission of meals or snacks was probed. Interviewers were trained in coding the diaries and could therefore identify the level of detail needed for different items. As fieldwork progressed, new codes were added for homemade dishes and items appearing in the diaries. Interviewers were asked to assess the quality of the dietary record and how far they thought it reflected the respondent’s normal diet. Further detailed checks were carried out for completion and consistency at the Office for National Statistics and then information from the dietary records was linked to the Nutrient Databank. This holds information on 56 nutrients, including water content, for each of the 6000+ food codes.

Respondents also completed a diary detailing the time spent on specified activities, and the intensity of activity, over the same 7 days as the dietary record. A physical activity score was calculated by multiplying the duration of each activity by the metabolic equivalent value (MET) for activities of that intensity. The average MET score represents an estimate of hourly energy expenditure per kg of body weight. Body weight was measured by trained personnel at the nurse visit after completion of diaries. Further details are given in Appendix D of the published report [22].

### Data preparation and analysis

Computerised raw data files and documentation from this survey, comprising weighed food records, questionnaires and biochemical and medical data, were obtained under license from the UK Data Archive (http://www.data-archive.ac.uk) and analysed using IBM® SPSS statistics v19 (SPSS IBM Inc Chicago, Illinois, USA).

Data were first analysed at the level of the individual food diaries, comprising weighed records for 12068 days (7 days for each survey respondent). Individual food items are recorded both with their original food codes and a higher level classification into 115 food groups for the published reports. The present analysis focused on total water intake (TWI) of all food and drink, as determined from food composition tables in the database. Metabolic water (water derived from oxidation of substrates) was not included as the focus was on comparison with dietary water requirements. Water in coffee or tea was coded as coffee/tea, while water for diluting concentrated soft drinks was coded as soft drink. Bottled water was coded separately from tap water. Milk includes all liquid milk, including that in tea and coffee, and milk consumed with cereal. Discretionary table sugar added to beverages was counted as food, rather than drink, as it was not possible to differentiate sugar use in drinks from sugar sprinkled on cereal or fruit consumed at the same time. For this project beverages were combined into 8 categories for further analysis: hot beverages (tea/coffee); milk; 100% fruit juice; caloric soft drinks (including sodas, juice drinks, sports and energy drinks); diet soft drinks; alcoholic drinks (including beer, cider, wine, spirits, alcopops); bottled water; and tap water. To investigate trends over the day, consumption occasions were aggregated into hourly intervals and into 5 periods, approximately corresponding to breakfast (<1000 hrs), lunch (>1000 to 1400 hrs), afternoon (>1400 to 1800 hrs), evening (1800 to 2200 hrs) and night (>2200 to 0200). Mean daily consumption, calculated over 7 days, was used to compare between individuals. TWI was compared with EFSA Dietary Reference Values (DRV) for Adequate Intake of Water (AI) for adult men and women of 2.5 L and 2.0 L, respectively [4]. The EFSA value for adequate intake is more conservative than other recommendations from WHO or IOM [5]. Nordic and German speaking countries take the approach that water intake is considered inadequate when it is less than 1 g per kilocalorie of energy requirement [4]. Therefore a combined classification (TWI-2), based on dual criteria, (i) the AI, and (ii) the ratio of water intake in grams to energy intake in kcal [4] was used to provide a more conservative estimate of the proportion of adults consuming low amounts of water (although with no implication of inadequate hydration status).

### Statistical analyses

Univariate analyses were carried out for men and women separately because of known differences in water consumption and recommended intakes. Crude differences in TWI and beverage consumption between groups were assessed using T-tests with Bonferroni correction for multiple comparisons. Non-parametric tests were used for variables that were markedly non-normal. Chi-square tests were used for categorical variables. All tests were 2-tailed and P < 0.05 taken as indicating statistical significance.

Multivariate analyses included adjustment for sex, age, bodyweight, smoking, dieting, physical activity level and mis-reporting (a recognized problem in dietary surveys with the potential to affect results) [23]. We attempted to adjust for implausible energy reporting using individuals’ ratio of energy intake (EI) to basal metabolic rate (BMR) [24] and applied Goldberg cut-offs corresponding to confidence limits of plausible intake based on a 7 day diet record at a physical activity level (PAL) of 1.55 [25]. Adults whose reported EI was <1.05 times their BMR were classified as under-reporters, while those with reported EI >2.28 times BMR were classified as over-reporters. Rather than exclude misreporters entirely, which can introduce bias, we included the classification variable in the analysis.

Analysis of covariance was used initially to examine how energy intake varied as the *proportion* of “liquid calories” increased (compared with food calories); covariates in the model were: sex, age, bodyweight, smoking, dieting, mis-reporting and physical activity. Multiple linear regression was used to estimate the effect on energy intake of varying the type of beverages consumed, whilst controlling for the effect of confounders. The effect of substituting non-caloric beverages by caloric beverages was estimated by including caloric beverages (as percentage of total beverage weight) as the main independent variable, with total beverage weight (g) held constant. This necessarily implies an equal and opposite change in other beverages. A further model included energy from food, thus disallowing compensation (reduction of food calories). Finally, we used the within-person daily consumption data to explore the effect of changes in consumption day-to-day, with each person acting as their own control. The independent variables in this analysis were all difference variables (deviation from the subject’s 7 day mean, deviation in total beverage consumption from subject mean) with deviation in energy intake as the outcome.

## Results

### Description of the survey sample

Survey respondents ranged from 19 to 64 years (mean 42 years), with slightly more women than men completing dietary records (Table 1). Around one third were current smokers and 25% of women (12% of men) were on a diet to lose weight. Obesity measures were representative of the UK adult population in 2000, with 60% overweight or obese.

**Table 1:** Descriptive statistics of the NDNS sample

			**Male**	**Female**	**Total**
		Count	766	958	1724
Age group					
	<35y	%	32%	33%	32%
	35 < 50y	%	39%	38%	39%
	50y+	%	29%	29%	29%
Social class					
	non-manual	%	53%	68%	61%
	manual	%	47%	32%	39%
Smoking habit					
	non-smoker	%	69%	67%	68%
	smoker	%	31%	33%	32%
On a diet to lose weight					
	No	%	89%	75%	81%
	yes	%	12%	25%	19%
Weight (kg)		Mean	84	69	76
		SE	1	1	0
Height (cm)		Mean	176	162	168
		SE	0.3	0.2	0.2
BMI - kg/m2		Mean	27.2	26.5	26.8
		SE	0.2	0.2	0.1
BMI class: normal, overweight or obese					
	normal weight	%	32%	46%	40%
	overweight	%	44%	33%	38%
	obese	%	24%	21%	22%

### Total water intake (TWI) and association with beverage consumption

**Figure 1:**
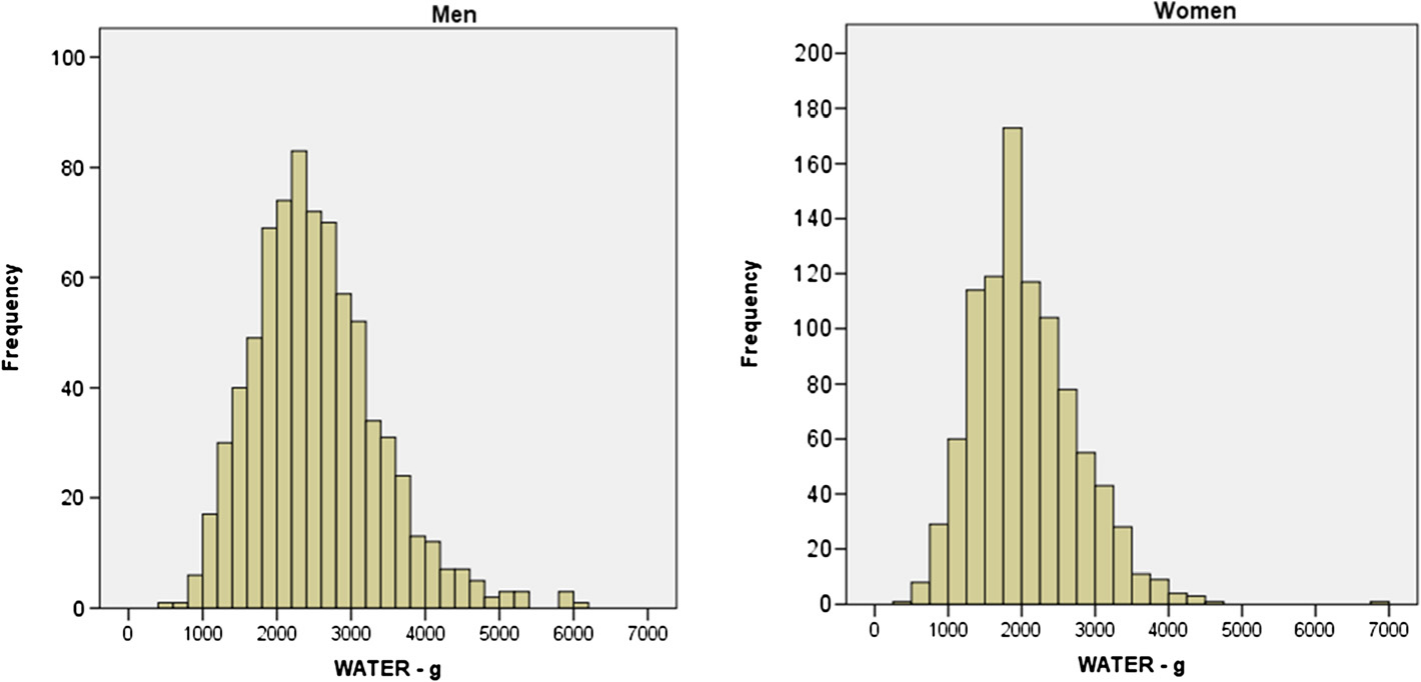
Frequency distribution of total water intake (g/d) over 7 days, by sex.

**Figure 2:**
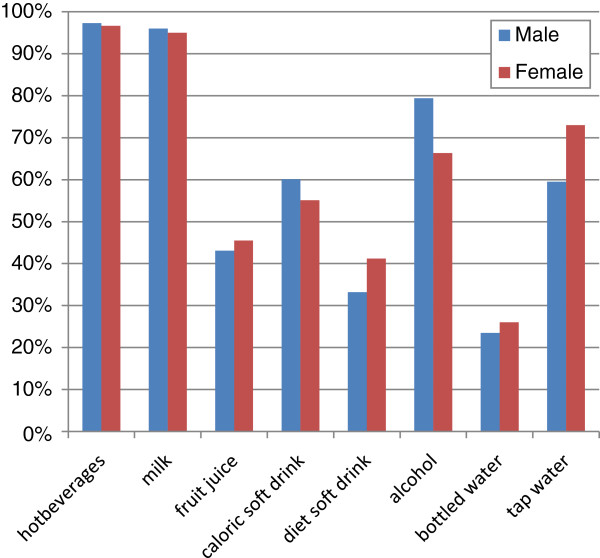
Popularity of beverages (% consuming over 7 day period).

Total water intake from all sources averaged 2.5 L and 2.0 L and men and women, respectively (Figure 1, Table 2). This concords with the estimated AI set by EFSA [4]. Beverages accounted for 75% of total water and 66% of the total weight of food and drink consumed. Mean beverage consumption was 1779 g/d (2012 g/d among men, 1593 g/d among women). Hot drinks (principally tea and coffee) were consumed by 97% of men and women, similarly for milk (95-96%) (Figure 2). Among men, alcoholic drinks were more popular than tap water (79% vs. 60% consumed, respectively), while 66% of women consumed alcohol and 73% drank tap water. More than half the sample drank caloric soft drinks at least once during the week, while less than half drank fruit juice, diet soft drinks or bottled water.

**Table 2:** Contribution of food and beverages to water and energy intake

		**Total weight consumed (g/d)**			**Contribution to water intake (g/d)**			**Contribution to energy intake (kcal/d)**		
		Male	Female	Total	Male	Female	Total	Male	Female	Total
	Count	766	958	1724	766	958	1724	766	958	1724
All food and drink	Mean	3055	2436	2711	2533	2059	2270	2305	1629	1929
	SE	34	24	22	31	23	20	22	14	15
Food only	Mean	1043	843	932	615	523	564	1907	1395	1622
	SE	11	9	7	8	6	5	20	13	13
Beverages only	Mean	2012	1593	1779	1918	1536	1706	399	234	307
	SE	30	21	19	29	21	18	9	5	5
hot beverages	Mean	774	708	737	765	702	730	16	11	13
	SE	18	15	12	17	15	12	1	1	1
milk	Mean	232	202	215	205	179	191	117	96	105
	SE	7	5	4	6	4	4	4	2	2
fruit juice	Mean	52	47	49	46	41	43	19	17	17
	SE	3	3	2	3	2	2	1	1	1
caloric soft drink	Mean	129	95	110	118	87	101	41	29	34
	SE	8	6	5	7	5	4	3	2	1
diet soft drink	Mean	82	93	88	81	93	88	1	1	1
	SE	7	7	5	7	7	5	0	0	0
alcohol	Mean	501	141	301	459	127	275	205	80	136
	SE	23	8	12	21	7	11	9	4	5
bottled water	Mean	55	63	60	55	63	60	0	0	0
	SE	6	6	4	6	6	4	0	0	0
tap water	Mean	187	243	218	187	243	218	0	0	0
	SE	13	11	9	13	11	9	0	0	0

Tea and coffee accounted for over 40% of the total daily weight of beverages while milk provided an additional 12% (232 g/d for men, 202 g/d for women) (Table 2). Men consumed much higher quantities of alcoholic drinks (mean 501 vs. 141 g/d for women; P < 0.0001) and slightly more caloric soft drinks (mean 129 vs. 95 g/d, P = 0.001). By contrast, women consumed more tap water (243 vs. 187 g/d for men; P < 0.0001) and diet soft drinks (93 vs. 82 g/d, P = 0.002). Bottled water and fruit juice were consumed in small amounts by both sexes (< 60 g/d). Younger men and women consumed less hot beverages and milk, but significantly more soft drinks (both caloric and non-caloric) and alcohol than older adults (Table 3). Mean consumption of caloric soft drinks over the total sample was 110 g/d or 2 cans per week, but younger women and men (<35y) consumed 140 g/d and 216 g/d, respectively. Consumption of alcoholic drinks in these groups averaged 179 g/d and 579 g/d, respectively. Beverages contributed approximately 16% of total energy overall (men 17%, women 14%) and alcohol was a major contributor (9% of EI for men; 5% for women) (Table 2).

**Table 3:** Total water intake and beverage consumption (g/d), by age group

		**Male**				**Female**			
		**age group**				**age group**			
		**<35y**	**35 < 50y**	**50y+**		**<35y**	**35 < 50y**	**50y+**	
		**A**	**B**	**C**	**Pairwise comparison**	**A**	**B**	**C**	**Pairwise comparison**
	Count	243	300	223		316	366	276	
Total Water intake from food and beverages	Mean	2443	2599	2543	ns	1895	2117	2170	C,B > A
	SE	58	49	54		38	36	44	
Water from food	Mean	555	617	677	C > B > A	450	527	600	C > B > A
	SE	12	11	16		9	10	13	
Water from beverages	Mean	1889	1982	1866	ns	1445	1590	1570	B > A
	SE	55	45	51		34	34	41	
Total beverage consumption (g)	Mean	1991	2077	1948	B > C	1503	1648	1622	B > A
	SE	58	47	53		34	34	42	
*of which (g/d)*									
hot beverages	Mean	594	813	916	C > B > A	512	784	833	C,B > A
	SE	29	28	32		23	24	30	
milk	Mean	217	237	240	ns	168	211	226	C,B > A
	SE	15	11	11		7	8	10	
fruit juice	Mean	43	60	53	ns	46	48	46	ns
	SE	5	6	6		4	5	5	
caloric soft drink	Mean	216	106	65	A > B,C	140	79	65	A > B,C
	SE	16	11	10		12	7	10	
diet soft drink	Mean	107	87	48	A > C	149	77	52	A > B,C
	SE	16	12	9		15	9	8	
alcohol	Mean	579	514	397	A > C	179	146	91	A,B > C
	SE	45	34	38		14	15	8	
bottled water	Mean	58	70	33	B > C	74	66	46	ns
	SE	11	11	6		12	10	8	
tap water	Mean	177	190	196	ns	234	236	263	ns
	SE	23	19	25		19	17	24	

Results are based on two-sided tests assuming equal variances with significance level 0.05.

Tests are adjusted for all pairwise comparisons within a row using the Bonferroni correction.

TWI was very highly correlated with the weight of beverages and water from beverages (r 0.97) and more weakly correlated with food intake (r 0.29) (Table 4). Hot beverages, tap water and alcohol were most highly correlated with TWI (r > 0.4). Alcoholic drinks (r 0.17), caloric soft drinks (r 0.15) and milk (r 0.14) had the highest correlation with EI, while for tap water and bottled water the coefficients were essentially zero.

**Table 4:** Partial Correlations between water intake, energy intake and beverage consumption, (7 day mean data, adjusted for age, gender, bodyweight, smoking, activity level, dieting, misreporting)

	Total water*	**Water from beverages**	**Water from food**	**Beverages weight**	**Food weight**	**Total energy kcal**	**Energy from beverages**	**Energy from food**
Total water*	1.00	.97**	.31**	.97**	.29**	.35**	.48**	1.00
Water from beverages	.97**	1.00	.07**	1.00**	.06*	.28**	.53**	.97**
Water from food	.31**	.07**	1.00	.06*	.96**	.32**	-.14**	.31**
Weight of Beverages	.97**	1.00**	.06*	1.00	0.04	.29**	.57**	.97**
Weight of food	.29**	.06*	.96**	0.04	1.00	.51**	-.20**	.29**
Total energy intake	.35**	.28**	.32**	.29**	.51**	1.00	.31**	.35**
Energy from beverages	.48**	.53**	-.14**	.57**	-.20**	.31**	1.00	.48**
Energy from food	.08**	−0.02	.40**	−0.03	.63**	.84**	-.25**	.08**
Hot beverages (g)	.48**	.50**	0.01	.49**	.05*	.11**	−0.01	.48**
Milk (g)	.25**	.26**	0.00	.27**	0.04	.21**	.27**	.25**
Fruit juice (g)	.09**	.06*	.13**	.06*	.11**	.08**	.11**	.09**
Caloric soft drink (g)	−0.01	0.01	-.08**	0.03	-.06*	.15**	.20**	−0.01
Diet soft drink (g)	.16**	.16**	.05*	.15**	.06*	.06*	−0.03	.16**
Alcohol (g)	.44**	.50**	-.12**	.52**	-.19**	.17**	.76**	.44**
Bottled water (g)	.21**	.18**	.14**	.18**	.12**	0.03	0.01	.21**
Tap water (g)	.43**	.40**	.18**	.39**	.16**	−0.01	-.05*	.43**
Variety of beverages consumed in day (out of 8)	.34**	.32**	.14**	.32**	.12**	.16**	.28**	.34**

* Water from food and beverages.

### Variety of beverages

Out of a maximum of 8 different types of beverages in our classification, the mean “variety score”, averaged over 7 days, was 3.3 for both men and women. Adults consuming fewer than 3 types had lower TWI and were less likely to meet the AI compared with those drinking 3 types or more (mean TWI 1.9 L vs. 2.4 L ; 73% vs. 43% below AI). Variety score was positively correlated with TWI (r 0.34, P < 0.0001; Table 4) and with EI (r 0.16, P < 0.0001), suggesting that beverage variety is an indicator of higher consumption of food and drink generally.

### Influence of day of the week

**Figure 3:**
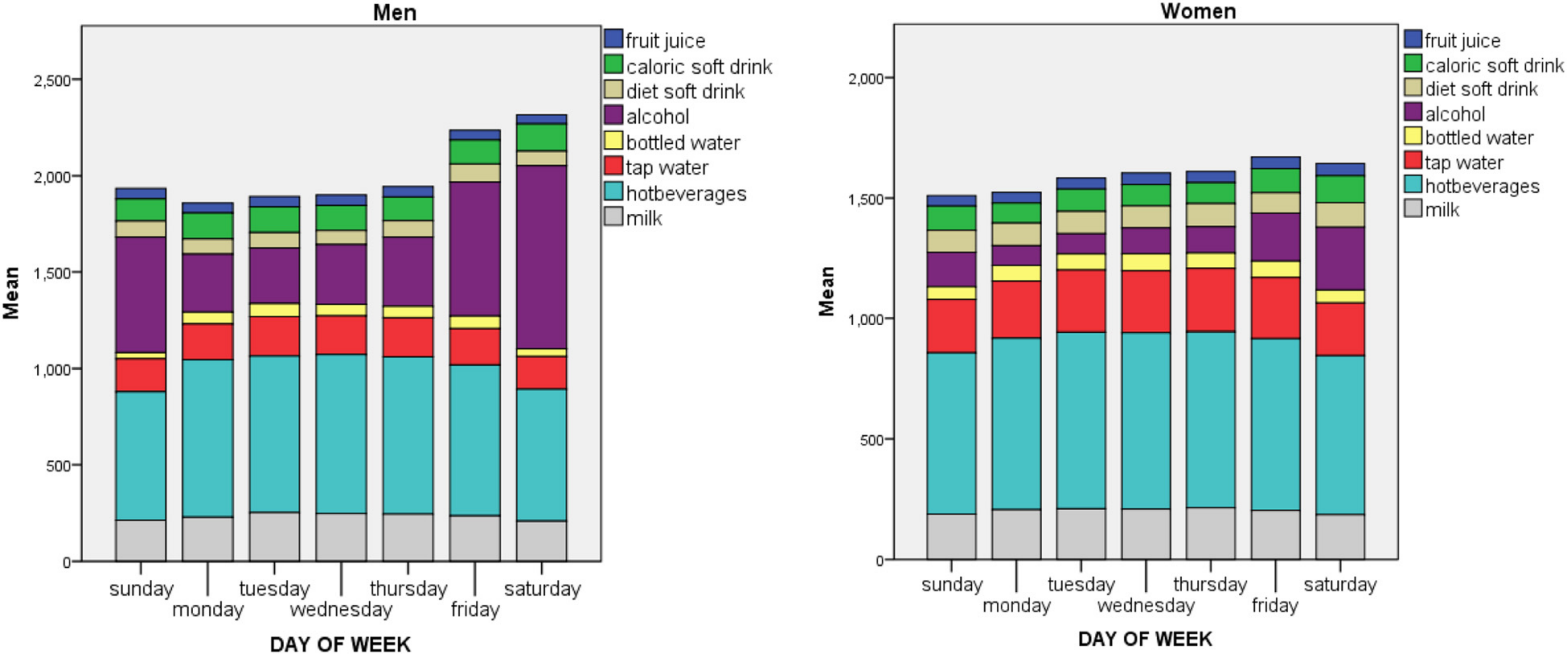
Amount and types of beverages consumed according to day of the week (mean g/d).

Total water intake and beverage consumption (g) was significantly higher on Fridays and Saturdays than on other days of the week, especially among men. This appeared to be attributable to higher consumption of alcoholic drinks at weekends, especially Saturday (Figure 3). Women showed weaker trends, but consumption on weekend days was still twice that on other days of the week. Saturday consumption of alcoholic drinks averaged 262 g among women and 949 g among men. Smaller differences in the opposite direction were observed for hot beverages and water. Consumption of soft drinks and fruit juice did not vary greatly by day of the week.

### Beverage consumption timeline over 24 hours

**Figure 4:**
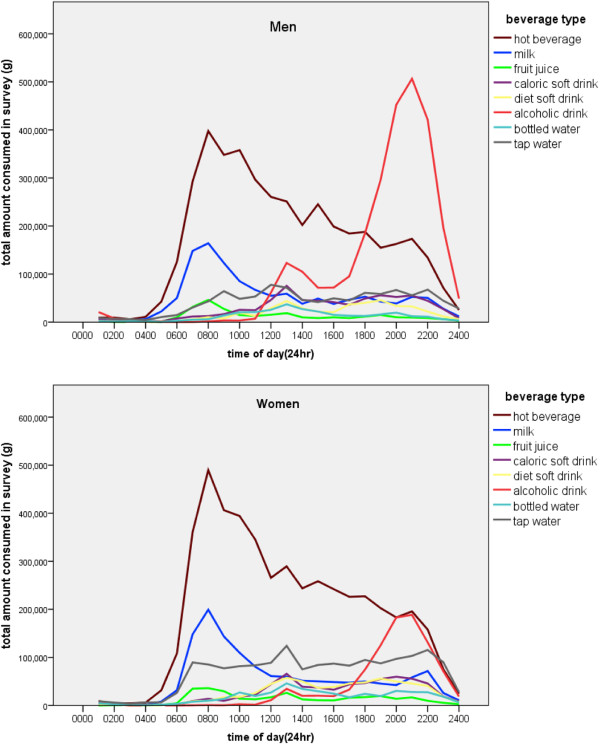
Time of consumption by beverage type.

Beverage consumption peaked at 0800 hrs and 2100 hrs for both men and women. When total consumption of each type of beverage was plotted as a separate line (Figure 4), a peak of hot beverages and milk in the morning and of alcohol in the evening was particularly noticeable, the latter being more marked in men. Other beverages were more evenly spread throughout the day, with slightly higher consumption around lunchtime (1300 hrs) and evening (1900 to 2100 hrs). Additional file 1: Appendix shows the 24 h time trends of beverage consumption for each day of the week (“24/7”).

Older adults consumed more beverages in the morning, young adults in the evening (Table 5). For example, men aged 50y + consumed on average 30% of their beverages before 1000 hrs, while young men (19-35y) consumed less than 22%; older men consumed 30% after 1800 hrs compared to 36% among younger men. Similar trends (all significant at p < 0.05) were observed among women. Evening consumption appeared to be driving the higher total beverage consumption on Fridays and Saturdays. Beverages consumed between 0200 hrs and 1000 hrs contributed 25% of total daily consumption and adults who drank nothing before 1000 hrs only partly compensated for the deficit, having a 16% lower beverage intake overall (data not shown).

**Table 5:** Beverage consumption according to time of day, by age group

	**Male**					**Female**				
	**Age group**					**Age group**				
**Mean amount of beverages (g/d) consumed between**	**<35y (A)**	**35 < 50y (B)**	**50y + ( C )**	**Total**	**Significant differences**	**<35y (A)**	**35 < 50y (B)**	**50y + ( C )**	**Total**	**Significant differences**
0200 to 1000 hrs	372	480	538	463	A < B < C	329	443	495	420	A < B < C
1000 to 1400 hrs	415	446	425	430	A < B	365	374	359	367	NS
1400 to 1800 hrs	365	381	339	364	C < B	289	321	319	310	A < B,C
1800 to 2200 hrs	648	580	509	581	A > B > C	399	406	343	386	A,B > C
2200 to 0200 hrs	191	190	137	175	A,B > C	121	104	106	110	A > B
Total Beverages	1991	2077	1948	2012	C < B	1503	1648	1622	1593	A < B,C

**Figure 5:**
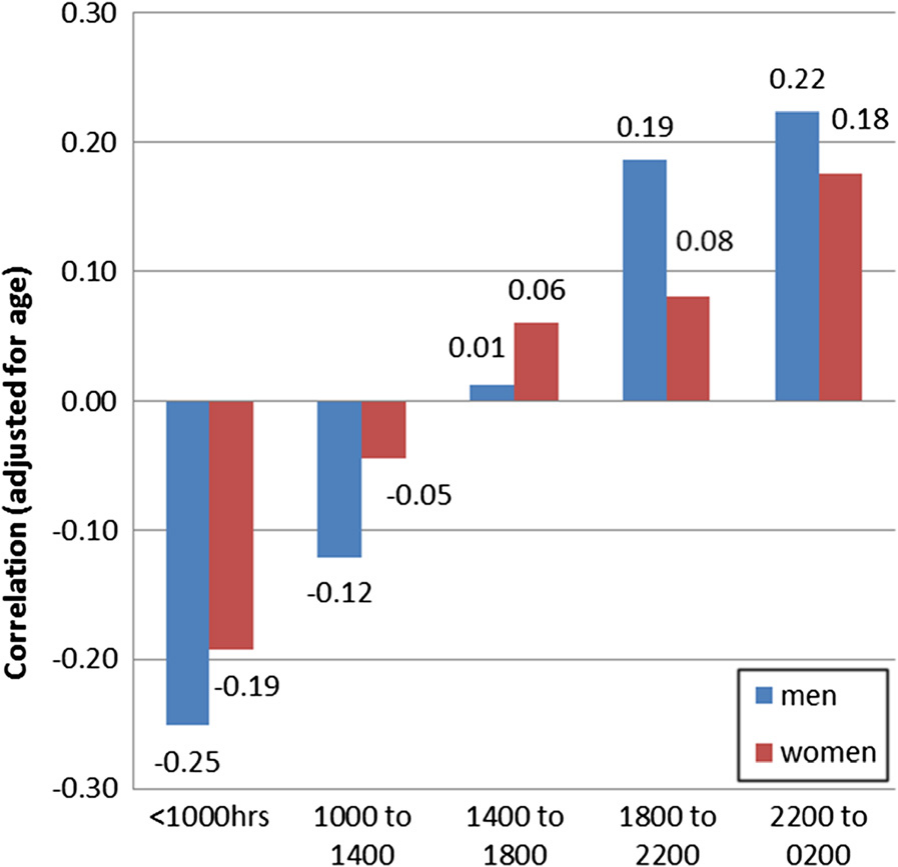
Correlation between total water intake and time of beverage consumption* (*beverage consumption in each period expressed as a percentage of total consumption over 24 hrs).

Adults with the highest TWI tended to consume most of their beverages in the evening. Figure 5 illustrates the correlation between timing of consumption (i.e. the proportion of beverage weight consumed in different time periods) and total water intake; the coefficients change from negative in the morning (r-0.24 for men, r-0.16 for women) to positive (r 0.22, r 0.17) for evening consumption. This effect is partly explained by the dominance of alcohol in evening, as (particularly for men) the correlation coefficients were attenuated when alcohol consuming days were excluded.

In conclusion, alcohol may be a major influence explaining the higher total water intake, higher evening consumption and higher weekend consumption of beverages observed in this study. On days alcohol was consumed, total water intake was 42% higher among men and 24% higher among women (data not shown).

### Combined classification for low vs. high water intake (TWI-2)

Men and women with water intakes below the EFSA AI (2.5 L or 2.0 L) who *also* had a low water to energy ratio (<1.0) were classified as having low water intake in both an absolute and a relative sense (low TWI-2), whilst those who were above the cut offs for both definitions were classified as high TWI-2. The remaining adults were excluded from the analysis. On this basis approximately 23% of all women and 33% of all men hade low TWI-2, while 45% and 41%, of men and women had high TWI-2. Men with high TWI-2 had slightly higher EI than men with low TWI-2 (2470 vs. 2287 kcal/d), but this was due to a higher contribution from alcohol (330 vs. 99 kcal/d) (P < 0.0001)(data not shown). For women, mean EI was not significantly different between the TWI-2 categories (1753 vs. 1716 kcal/d; P = 0.32). On a proportional basis, women with low TWI-2 drank significantly less water and hot beverages, while men consumed less water and alcohol compared with the high TWI-2 adults. Adults with low TWI-2 had diets of higher energy density and this applied to foods as well as beverages.

### Estimated effect of caloric beverages (liquid calories)

There was no difference in total energy intake when beverage calories increased at the expense of food calories (Table 6); (i.e. “a calorie is a calorie”). This is one interpretation of the liquid calories hypothesis. However, a more relevant question for public health is to what extent replacing caloric beverages by non-caloric beverages would help reduce energy intake and (possibly) reduce obesity.

**Table 6:** Total energy intake according to relative contribution of beverages vs. food

		**%energy from beverages (quintiles)**				
		**<= 9**	**10-12**	**13 - 16**	**17 - 21**	**22+**
ENERGY - kcal	mean	1953	1940	1941	1969	1949
	SE	20	20	19	19	20

(estimated marginal means, adjusted for covariates: age Sex, current smoker, weight, physical activity, mis-reporting , dieting status).

To address this, our first models (Table 7) included 7 covariates (sex, age, bodyweight, smoking, dieting, physical activity and misreporting) and also total beverage intake, hence mathematically equivalent to substituting caloric beverages (sum of milk, fruit juice, caloric soft drinks and alcoholic beverages) for an equal weight of non-caloric beverages (hot beverages excluding milk, diet drinks, tap water and bottled water). The predicted effect of replacing 100 g caloric beverages with non-caloric drink was a reduction of 15 kcal (allowing food intake to vary) (Table 7, Model 1). When food energy intake was constrained as constant (i.e. disallowing compensation) the net impact of 100 g caloric beverages was estimated at 34 kcal (Table 7, Model 2). These estimates are based on the consumption patterns of other adults in the survey with similar patterns.

**Table 7:** Regression model estimating change in energy intake associated with adding caloric beverages in place of non-caloric beverages

**Model 1**				
	**B**	**P value**	**95% Confidence interval for B**	
			**Lower**	**Upper**
(Constant)	1040	<0.0001	852	1227
Sex -female	−371	<0.0001	−414	−328
Age (years)	−1.6	0.044	−3.1	0.0
current smoker	−63	0.001	−100	−25
weight (kg)	7.2	<0.0001	6.0	8.4
Dieting	−67	0.005	−114	−20
Activity (MET)	147	<0.0001	104	190
Valid reporter	659	<0.0001	617	700
Total beverages (100 g/d)	11	<0.0001	10	12
4caloricbeverages (100 g)*	15	<0.0001	10	20
**Model 2, Keeping Food Constant**				
	**B**	**P value**	**95% Confidence Interval for B**	
			**Lower**	**Upper**
(Constant)	62	0.01	15	109
Sex -female	−20	0.001	−31	−8
Age (years)	0.4	0.023	0.6	0.8
current smoker	16	0.001	7	26
weight (kg)	0.2	0.241	−0.1	0.5
Dieting	1	0.826	−10	13
Activity (MET)	−4	0.513	−14	7
Valid reporter	40	<0.0001	28	53
Food (kcal)	0.97	<0.0001	0.96	0.98
Total beverages (100 g)	2	<0.0001	1	3
4caloricbeverages (100 g)*	34	<0.0001	33	36

* 4caloric beverages = sum of milk, fruit juice, caloric soft drinks and alcoholic drinks.

The second analysis (Table 8) used a within-person change model to address whether a change in an individual’s beverage consumption habit on any day was associated with a change in their total energy intake (compared with their 7 day mean). Modelling each beverage separately, with total beverage weight held constant, each of the 4 non-caloric beverages was negatively associated with energy, while the 4 caloric beverages were positively associated with energy (Table 8, Model 3). Combining caloric beverages together, the final models (Table 8, Models 4 & 5) gave an estimated effect of 29 kcal per 100 g of caloric drinks substituted, or 35 kcal if food energy (kcal) was held constant. By comparison, the mean caloric content of the caloric beverages consumed (calculated from the database) was 47 kcal/100 g.

**Table 8:** Within–person change Model: estimated change in energy intake associated with beverage substitution

Model 3 Estimated effect of substituting each beverage type *				
	**B**	**P value**	**95% Confidence interval for B**	
**Change in:**			**Lower**	**Upper**
Hot beverages (100 g)	−15	<0.0001	−18	−12
Milk (100 g)	15	<0.0001	8	22
Fruit juice (100 g)	25	<0.0001	15	35
Caloric soft drink (100 g)	23	<0.0001	18	27
Diet soft drink (100 g)	−15	<0.0001	−20	−9
Alcohol (100 g)	17	<0.0001	15	19
Bottled water (100 g)	−21	<0.0001	−27	−15
Tap water (100 g)	−31	<0.0001	−35	−28
**Model 4 Estimated effect of caloric beverages replacing non-caloric beverages**				
	**B**	**P value**	**95% Confidence Interval for B**	
			**Lower**	**Upper**
Change in total beverages (100 g)	12	<0.0001	10	14
Change in caloric beverages (100 g)	29	<0.0001	27	32
**Model 5 Estimated effect of caloric beverages replacing caloric beverages, holding food energy constant**				
	**B**	**P value**	**95% Confidence Interval for B**	
			**Lower Bound**	**Upper Bound**
Change in food energy (100 kcal)	100	<0.0001	99.9	101
Change in total beverages (100 g)	1	<0.0001	0.9	1.8
Change in caloric beverages (100 g)	35	<0.0001	35	36

*Model 3 is a composite of 8 regressions, one for each beverage, adjusted for change in total weight of beverages; coefficients express the change in energy intake associated with a change in one beverage type and a corresponding reduction in others, i.e. constant total beverage intake.

## Discussion

Using 12068 days of weighed dietary records from a nationally-representative sample of British adults aged 19 to 64 years, we have quantified total water intakes, and investigated relationships between beverage consumption patterns and water and energy intakes.

Mean TWI was almost identical to the European (EFSA) reference or “adequate intake” (AI) of 2 L for women and 2.5 L for men [4]. EFSA determined their AIs from a combination of observed intakes in population groups, with desirable urine osmolarity values and desirable water volumes per energy unit consumed. These AIs only apply to conditions of moderate environmental temperature and moderate physical activity levels; physically active individuals or those working in hot conditions may require more. However, AIs are likely to exceed requirements and cannot predict which individuals are under- or over- hydrated in practice. Using more conservative criteria (TWI below AI *and* TWI <1 g per kcal of energy intake) approximately 33% of men and 23% of women were classified as having low water intakes. These may be at greater theoretical risk of poor hydration, but may actually be appropriately hydrated if they have low volumes of water loss due to their individual physiology, environment and physical activity. In our opinion a value of 1 g water per kcal energy requirement (rather than energy intake) would seem an appropriate basis for a UK recommended mean water intake, and has the utility of broad applicability across age/sex groups. However, it would be helpful to attempt to specify a confidence interval around this mean to avoid confusion and misuse.

Beverages supplied 75% of TWI for adults in this British study. This is consistent with other estimates [4,7,8], but the proportion may vary considerably between individuals and between populations. As we have shown, beverage consumption (and water intake) is not evenly spread throughout the day, but tends to be concentrated in the evening. This leaves the possibility that some adults may be relatively overhydrated in the evening/nighttime and under-hydrated in the morning. As alcoholic beverages were a significant source of water and energy, especially among men, and were strongly associated with evening consumption, men who drink alcohol may be particularly vulnerable. TWI can be increased by offering more variety in beverages [26]. However, if such an intervention is to benefit public health, it is important that the additional fluid is consumed when needed, and does not result in excessive water intake, unwanted effects on energy balance, or inadequate nutrition.

Since our analysis was conducted, Ng et al. [9] have reported on UK trends in beverage consumption over the past 25 years. There are some differences in methodology between our studies, but their calculation for adults in 2000/2001 (18% of energy obtained from beverages) compares with our estimate of 16% (which excluded discretionary sugar). Had we been able to include table sugar added to drinks, we would have seen a small contribution of tea and coffee to energy intake but our overall findings would not have been affected. Between 2000 and 2008/9 the energy intake contributed by beverages (in total) did not change, while sweetened tea and coffee declined and consumption of alcoholic beverages and caloric soft drinks rose, although not significantly [9]. In 2008/9 alcoholic beverages provided 770 kJ/d (184 kcal/d) per capita, and caloric soft drinks (soda and juice drinks) 209 kJ (50 kcal/d) per capita; hence our conclusions concerning the dominance of alcohol appear to remain valid. According to Ng et al., TWI increased by approximately 9% between 2000/2001 and 2008/2009, as people consumed more water as a beverage and more water in food (fruit and vegetables) [9], a trend that may have been influenced by healthy eating campaigns and marketing of bottled water. However, consumption of water, tea and coffee, soft drinks and juice among adults has changed little over the last 3 years (NDNS 2008–2011) [27,28], and plain water consumption in the UK remains lower than among adults in France [11], Canada [29] or America [30]. Consumption habits are particularly well- documented in the US, and NHANES surveys show alcohol providing (only) 115 kcal/d for adults, while soda /fruit drinks provided 141kcaL/d in 2005/2006 [30]. It appears that the main increase in soft drink consumption in the US occurred between the 1970s and the 1990s, and a new study confirms that liquid calories have been falling in the US diet over the past decade, although consumption remains high [31].

The present study demonstrates that well-conducted national surveys such as the NDNS have the potential to yield rich contextual data that can be linked with nutrition and health measures. We found significant variation in consumption by day of the week and by time of day (see Additional file 1: Appendix for supplementary figures), which few other studies have been able to describe in detail. Alcoholic drinks in particular were a major contributor to evening and weekend peaks in beverage consumption. The extent to which timing of drinking occasions relates to overall TWI and EI requires further study. Associations have also been demonstrated between meal patterns and EI, with the suggestion that evening consumption of calories may be more conducive to total energy excess [32]. However these findings may merely reflect the cultural reality of the evening being a time for eating and drinking. Significantly, and possibly for the first time using such data, we have estimated the effect on EI of switching beverage consumption between caloric and non-caloric sources using within-person daily records.

In terms of the limitations of this study we draw attention to the usual caveats on making causal inferences from observational data. Whilst the data are of the highest quality obtainable, and respondents were revisited to check records and probe for missing items and weights, dietary assessment is an imperfect science. Most errors are likely to be in the direction of omission or underestimation (perhaps especially where alcohol is concerned). Errors of overestimation may also have occurred where a drink was spilt or unfinished, although efforts were made to prevent this by asking respondents to weigh leftovers and estimate spills. There is evidence from validation studies that true EI is underestimated by about 20-25% in the NDNS [33], but to our knowledge no studies have tried to quantify under-reporting of water intake or beverages specifically. Estimates of water intake adequacy based on survey data are likely to suffer from under-estimation also.

In conclusion, a significant proportion of British adults surveyed in 2000/2001 had low TWI, although in the absence of clinical measures this could not be equated with poor hydration status. Further work may be warranted to assess current intakes and to explore correlations with urine volumes, as recommended recently [3]. Soft drinks were not a major source of liquid calories among these British adults, whereas alcohol was more significant. All beverages supplying energy can contribute to higher total EI, but in practice some degree of compensatory under-consumption of other foods or beverages may occur. Mechanistic and experimental studies are required to address directly the satiety effects of beverages, while work is also needed to understand the drivers of beverage consumption, which are not merely physiological but also psychological, social and environmental. Further research based on dietary patterns rather than singular foods or dietary components may help identify unhealthy behaviors and provide better evidenced-based recommendations for adequate fluid intake and optimal beverage consumption.

## Competing interests

SG has received funding for research and consultancy from a number of food and beverage companies. SS has received funding for research and consultancy from a number of companies that produce drinks.

## Authors’ contributions

SG conceived the project and was responsible for analyzing and interpreting data, drafting and finalizing the paper. SS contributed to interpretation of results and discussion. Both authors read and approved the final manuscript.

## Supplementary Material

Additional file 1: Appendix Time charts of beverage consumption for each day in males and females. Click here for file
