# Supplementary figures and images for: Beverage consumption habits “24/7” among British adults: association with total water intake and energy intake
Source: Nutr J. 2013 Jan 10;12:9. doi: 10.1186/1475-2891-12-9 (PMC3575376; doi:10.1186/1475-2891-12-9)

**Appendix.**

**Time charts of beverage consumption for each day in males and females**


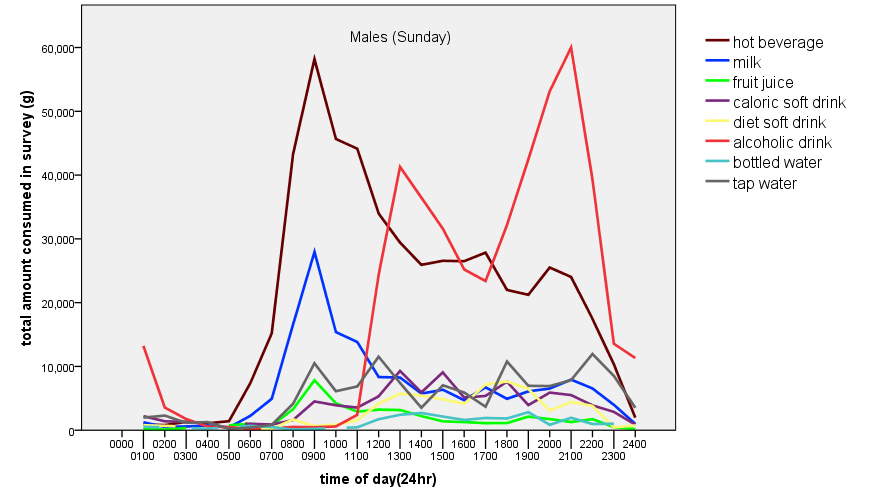


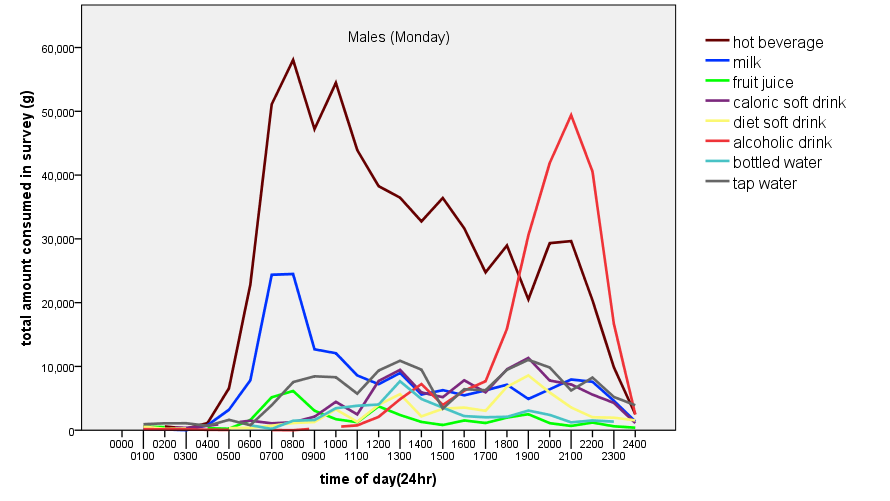


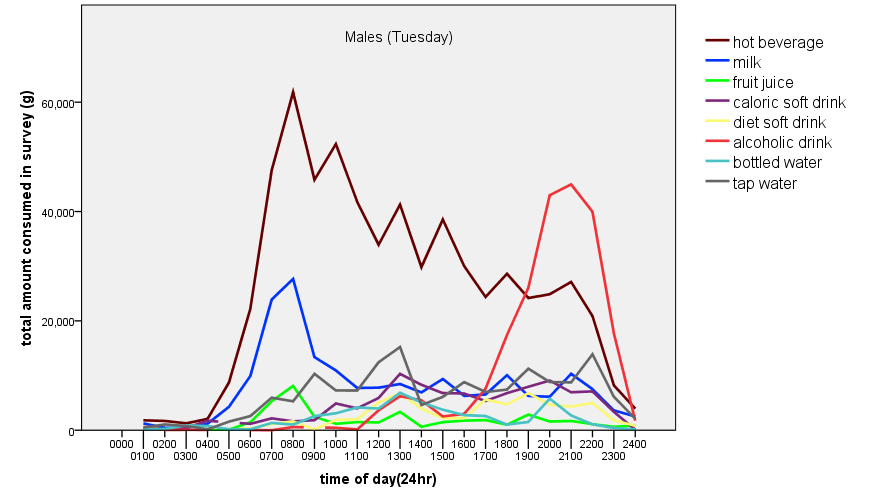


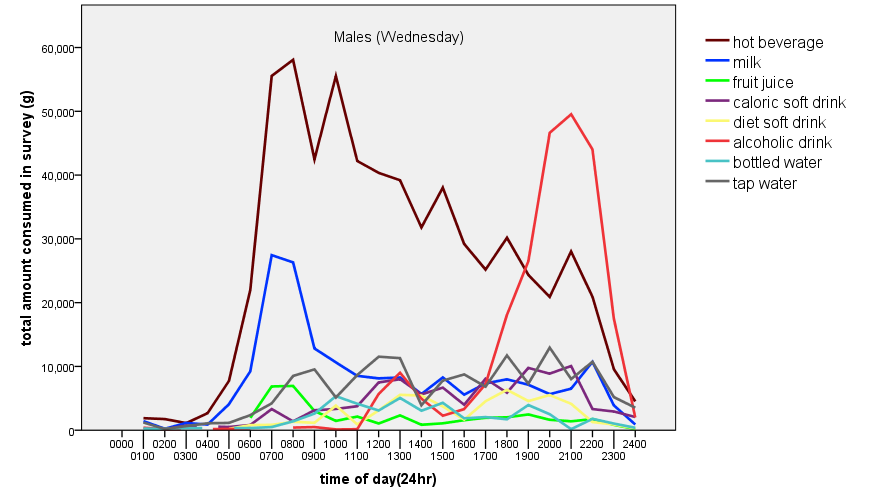


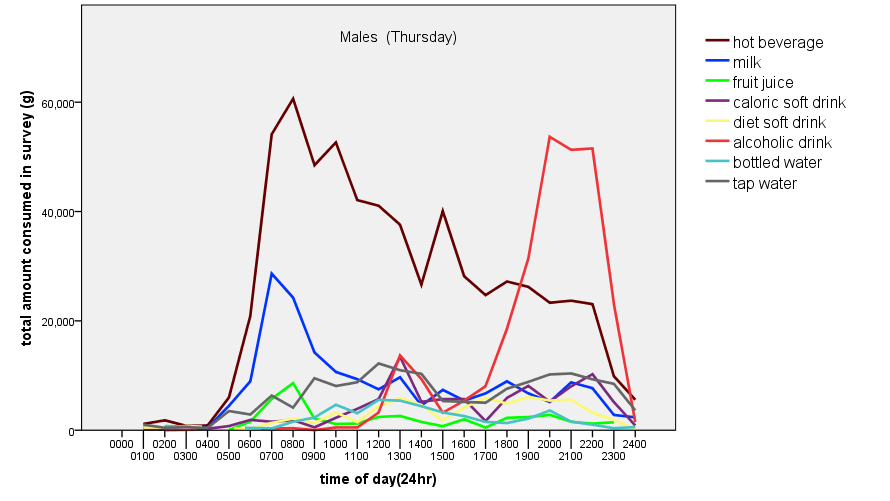


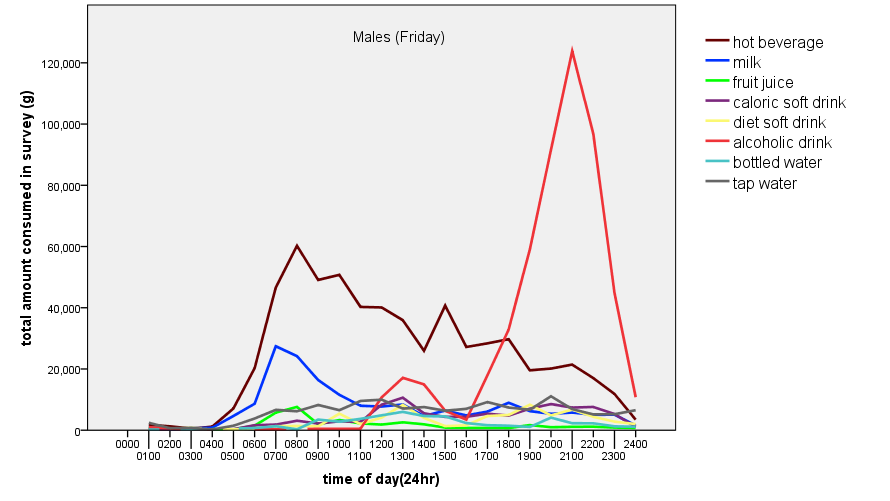


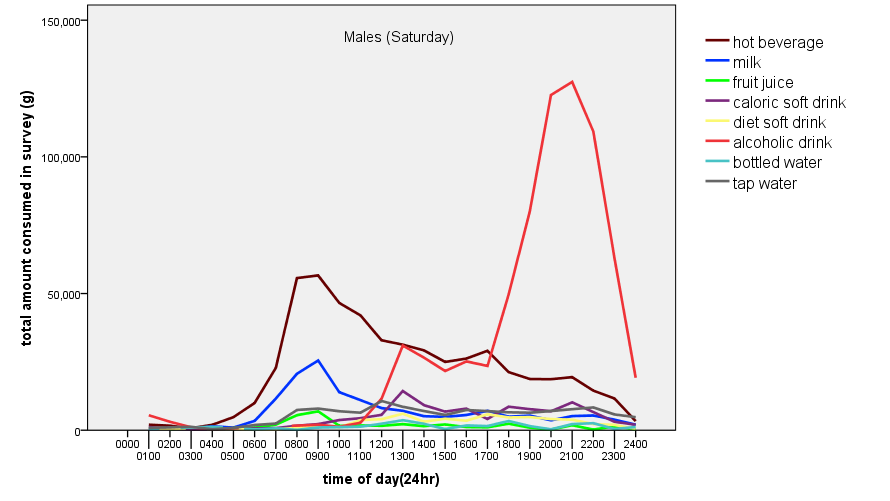


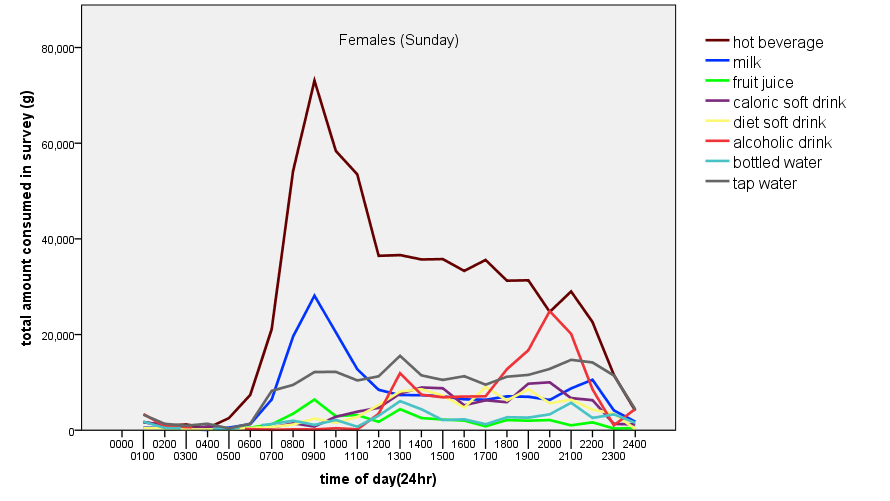


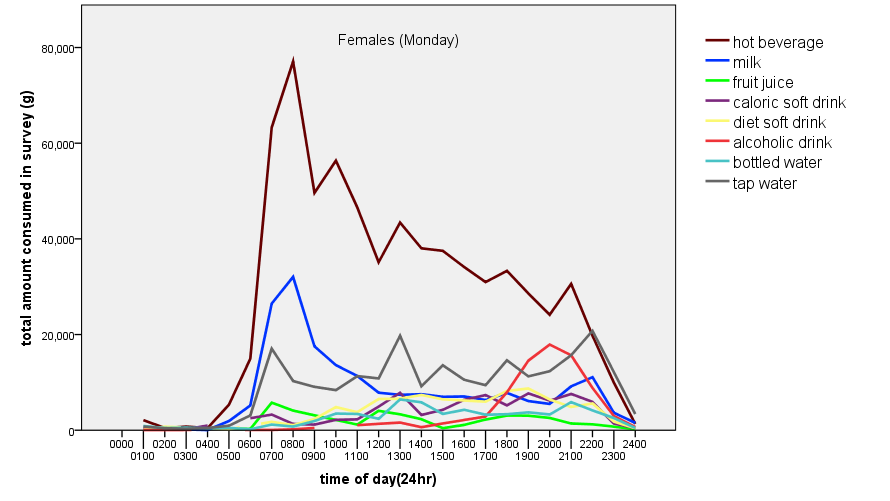


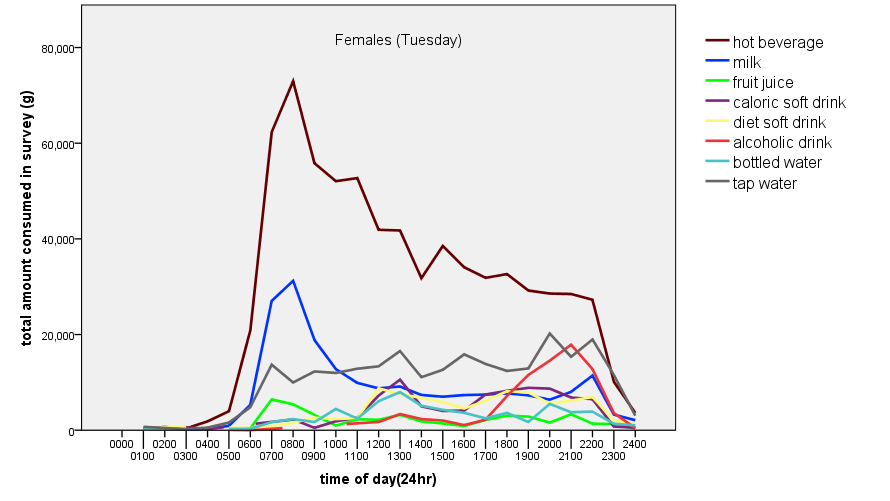


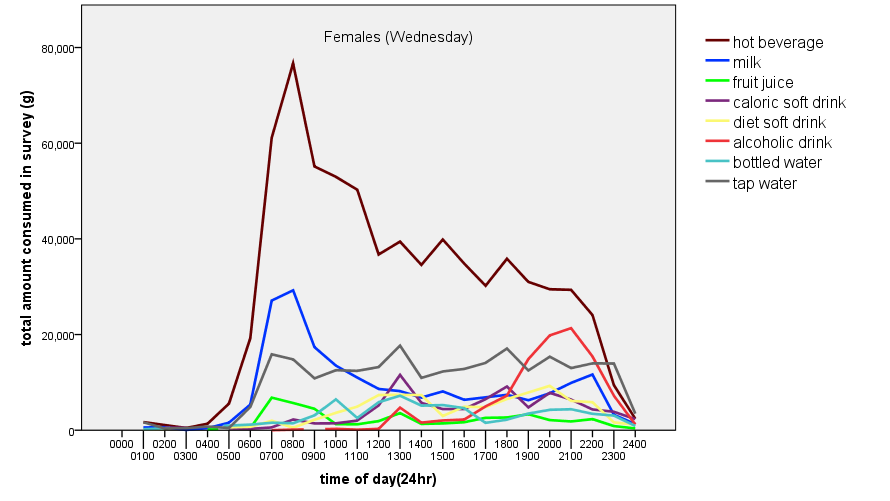


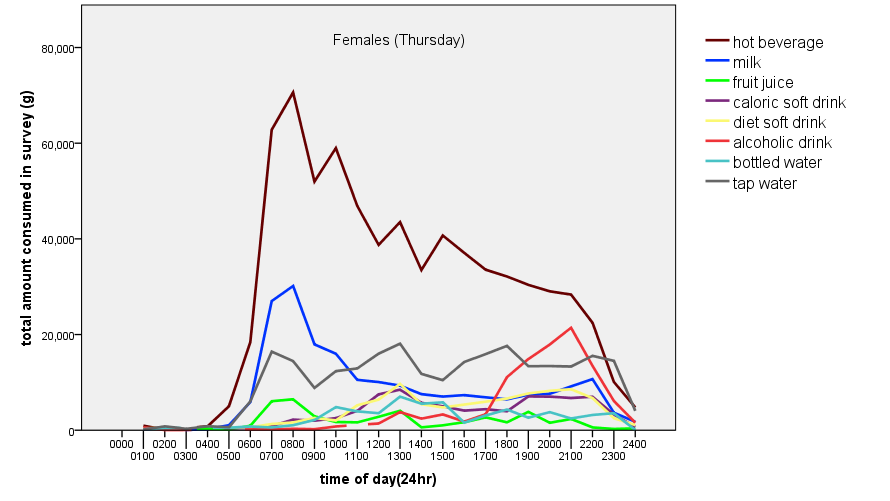


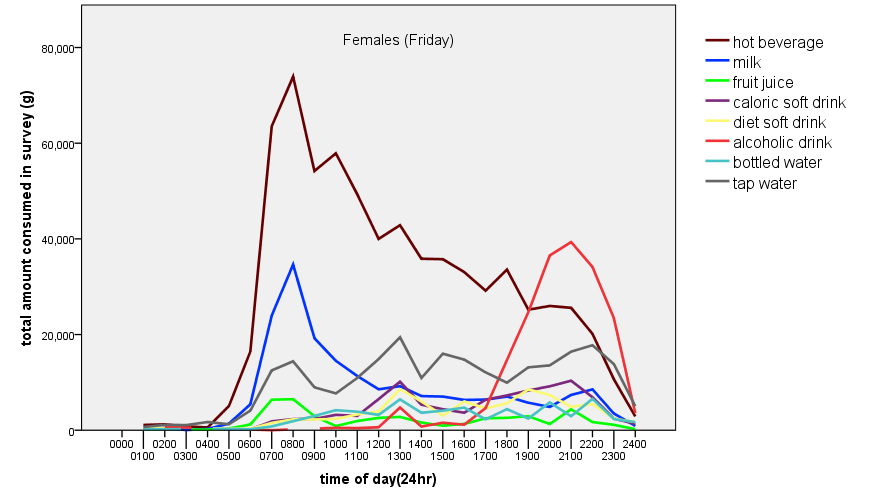


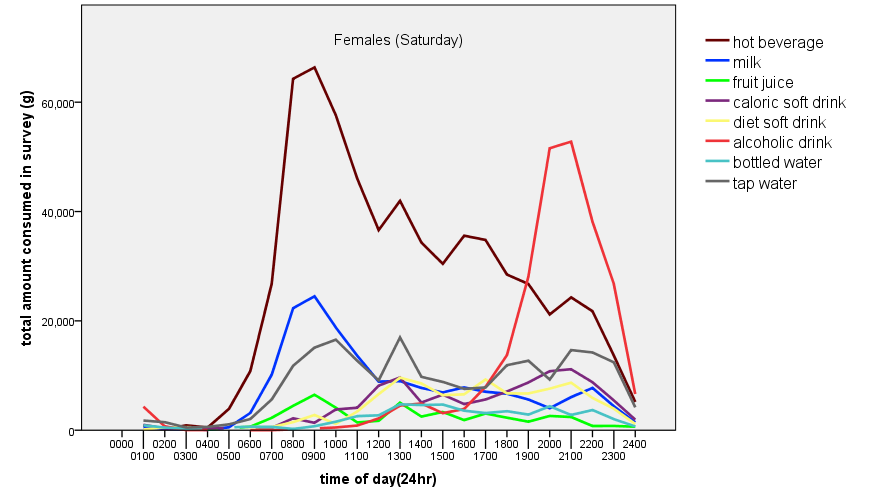

Supplement: Additional file 1: Appendix — Time charts of beverage consumption for each day in males and females. [file 1475-2891-12-9-S1.doc]
